# Volcanic influence on centennial to millennial Holocene Greenland temperature change

**DOI:** 10.1038/s41598-017-01451-7

**Published:** 2017-05-03

**Authors:** Takuro Kobashi, Laurie Menviel, Aurich Jeltsch-Thömmes, Bo M. Vinther, Jason E. Box, Raimund Muscheler, Toshiyuki Nakaegawa, Patrik L. Pfister, Michael Döring, Markus Leuenberger, Heinz Wanner, Atsumu Ohmura

**Affiliations:** 1 Climate and Environmental Physics, University of Bern, 3012 Bern, Switzerland; 2 Oeschger Centre for Climate Change Research, University of Bern, 3012 Bern, Switzerland; 3 Climate Change Research Centre and PANGEA Research Centre, University of New South Wales, New South Wales, 2052 Australia; 4 ARC Centre of Excellence for Climate System Science, New South Wales, Sydney Australia; 5 Centre for Ice and Climate, Niels Bohr Institute, University of Copenhagen, 2100 Copenhagen, Denmark; 6 Geological Survey of Greenland and Denmark, 1350 Copenhagen, Denmark; 7 Department of Geology, Quaternary Sciences, Lund University, 22362 Lund, Sweden; 8 Meteorological Research, Institute, Tsukuba, 305-0052 Ibaraki, Japan; 9 Institute for Atmospheric and Climate Science, Swiss Federal Institute of Technology ETH Zurich, 8092 Zurich, Switzerland; 10 Present Address: Renewable Energy Institute, Minato-ku, 105-0003 Tokyo, Japan

## Abstract

Solar variability has been hypothesized to be a major driver of North Atlantic millennial-scale climate variations through the Holocene along with orbitally induced insolation change. However, another important climate driver, volcanic forcing has generally been underestimated prior to the past 2,500 years partly owing to the lack of proper proxy temperature records. Here, we reconstruct seasonally unbiased and physically constrained Greenland Summit temperatures over the Holocene using argon and nitrogen isotopes within trapped air in a Greenland ice core (GISP2). We show that a series of volcanic eruptions through the Holocene played an important role in driving centennial to millennial-scale temperature changes in Greenland. The reconstructed Greenland temperature exhibits significant millennial correlations with K^+^ and Na^+^ ions in the GISP2 ice core (proxies for atmospheric circulation patterns), and δ^18^O of Oman and Chinese Dongge cave stalagmites (proxies for monsoon activity), indicating that the reconstructed temperature contains hemispheric signals. Climate model simulations forced with the volcanic forcing further suggest that a series of large volcanic eruptions induced hemispheric-wide centennial to millennial-scale variability through ocean/sea-ice feedbacks. Therefore, we conclude that volcanic activity played a critical role in driving centennial to millennial-scale Holocene temperature variability in Greenland and likely beyond.

## Introduction

Holocene climate variability is important to understand the process of human societal development from a hunter-gatherer society to the present complex society^1,2^. However, precise understanding of Holocene climate variability on multidecadal to millennial scales has been elusive owing to the lack of adequate archives recording small temperature signals and poorer chronologies further back in time^3^. Therefore, we employed a relatively newly established method of temperature reconstruction using argon and nitrogen isotopes in occluded air within ice cores (Methods; Figs S1 and S2)^4^. This method relies on temperature-dependent gas fractionation in the unconsolidated snow layer^5^. Temperature gradients between the top and bottom of the unconsolidated snow layer induce gas fractionation^6^. After which the fractionated gasses are trapped in ice at the bottom of the firn layer^4^. By measuring nitrogen and argon isotopes in ice cores, we can reconstruct past temperature gradients (Fig. S3)^4^, which combined with observed borehole temperature data and a firn densification/heat diffusion model, are used to reconstruct past surface temperature changes^4,7,8^. Reconstructed temperatures (Supplementary Dataset) are seasonally unbiased multidecadal average temperatures due to the processes of heat and gas diffusion in the firn layer^4,5^, and are constrained by borehole temperature profiles^4^ (Method; Fig. S4). Sampling density is not constant through the Holocene such that uncertainty ranges of the reconstructed temperatures vary with time^9^.

**Figure 1:**
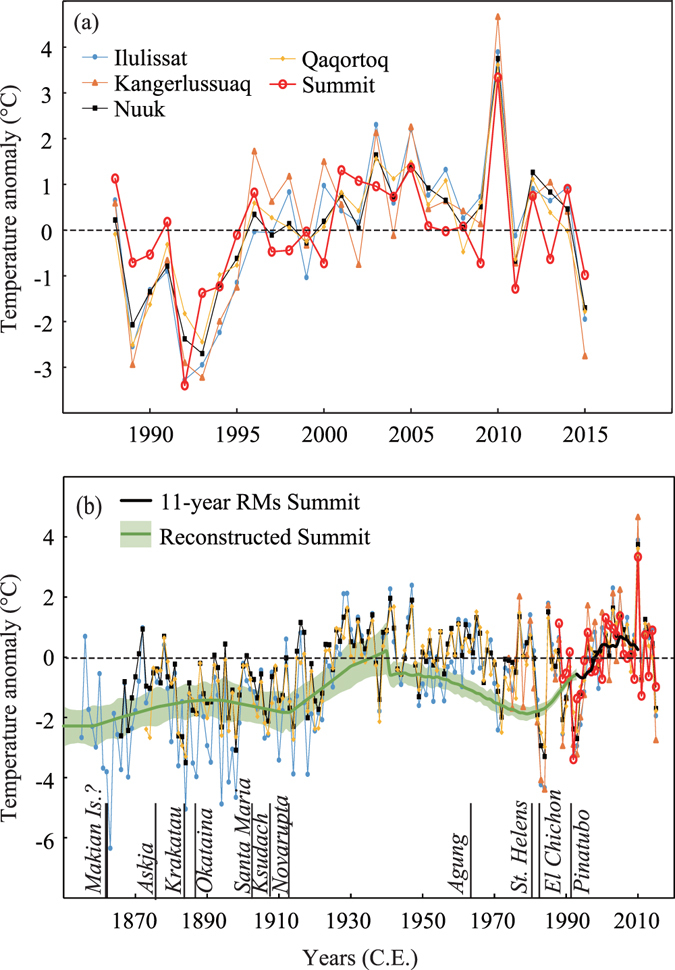
Greenland temperature anomalies relative to averages of 1988–2015 at the Summit and four coastal stations. (**a**) Temperature anomalies from 1988 to 2015. (**b**) Same as (**a**) but from 1850 to 2015. Green line with 2σ error bands is the reconstructed temperature anomaly. The reconstructed temperature (snow temperature) was adjusted to have the same value in 1993 as an average of the observed Summit temperature (air temperature) for 1988–1998 by adding 1.0 °C. The names and timings of large volcanic eruptions are according to Box *et al*.^13^.﻿ RMs represents running means.﻿

Continuously observed instrumental Greenland Summit air temperature (GISP2) is now available for 28 years^10^ (1988 to 2015; Fig. 1a), allowing us to place the current decadal average temperature into the long-term context of the Holocene. The record exhibits the characteristic temperature drop in 1992–1993 associated with cooling caused by the eruption of Mt. Pinatubo^11^, followed by an increase until 2005 (Fig. 1a). For the most recent 10 years (2005 to 2015), apart from the anomalously warm year of 2010, mean annual temperatures at the Summit exhibit a slightly decreasing trend in accordance with northern North Atlantic-wide cooling^12^. The Summit temperatures are well correlated (*r* ~ 0.7–0.8; *p* < 0.01) with southwest coastal records (Ilulissat, Kangerlussuaq, Nuuk, and Qaqortoq; Fig. 1a). The high correlations between the Summit record and coastal temperatures allow us to use the longer coastal records to estimate Greenland Summit temperatures over the past 160 years (Fig. 1b). During this period, large volcanic eruptions caused 1 to 3 years cooling episodes in Greenland^13^. Importantly, the reconstructed nitrogen-argon-isotope-based temperatures (hereafter, reconstructed temperatures) agree well with the instrumental records on a multi-decadal scale (Fig. 1b).

**Figure 2:**
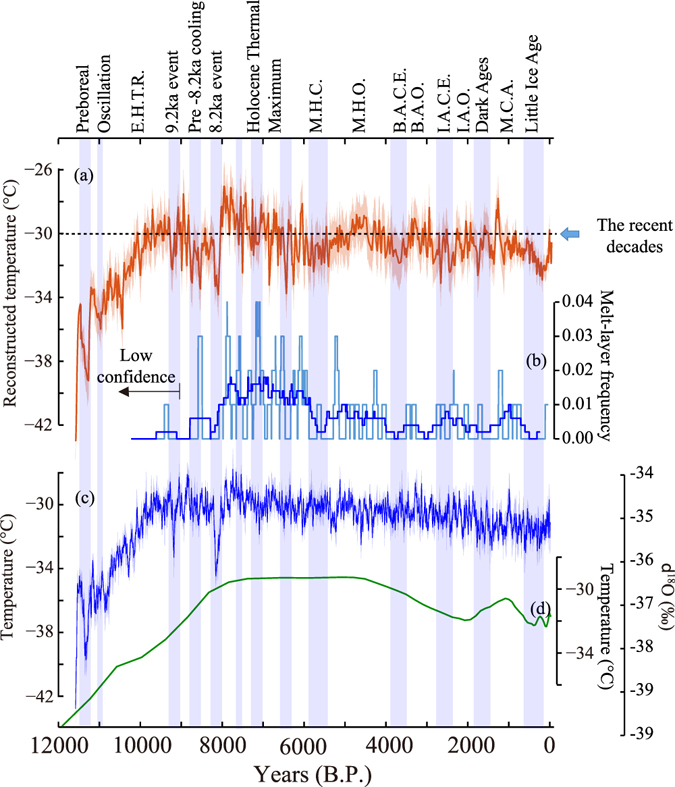
Greenland Summit temperature and its proxies over the Holocene. (**a**) Reconstructed temperature from argon and nitrogen isotopes with 2σ error bands. (**b**) Melt-layer frequency (times per years) in 100 and 500-year RMs with light blue and blue lines, respectively. Note that the data before 9000 B.P. has low confidence^20^. (**c**) δ^18^O_ice_ and a calibrated temperature scale in 20-year RMs with 2σ error bands from GISP2^14^, GRIP, and NGRIP^15^ (Methods) (**d**) GRIP borehole temperature inversion^42^. Blue shades are Greenland cold episodes. E.H.T.R. = Early Holocene Temperature Rise, M.H.C. = Mid-Holocene Cooling, M.H.O. = Mid-Holocene Optimum, B.A.C.E. = Bronze Age Cold Epoch, B.A.O. = Bronze Age Optimum, I.A.C.E. = Iron Age Cold Epoch, I.A.O. = Iron Age Optimum, M.C.A. = Medieval Climate Anomaly. The names are given according to the Greenland temperature changes or common usages if available.

The reconstructed temperatures exhibit a long-term trend similar to that of average δ^18^O_ice_ (GISP2^14^, GRIP and NGRIP; Fig. 2). However, the δ^18^O_ice_ records lack strong centennial to millennial-scale variability (e.g., the Medieval Climate Anomaly and Little Ice Age; Fig. 2). We find that 27% of the Holocene Greenland temperatures are higher than the present multi-decadal average (1988–2015, hereafter “the recent decades”; Figs 1 and 2a, Fig. S5). The reconstructed temperature reached the present level around 9,500 years B.P., which is earlier than that of the borehole based reconstruction (around 8,000 years B.P., Fig. 2). This is likely because borehole temperature reconstructions are unable to capture sharp transitions such as the one from the cold Younger Dryas to the warm Holocene due to the smoothing effect by diffusion of heat in the ice-sheet (Methods). Consistent with an earlier conclusion^14,15^ from δ^18^O_ice_, Greenland climate remained relatively stable and warm during the Holocene, compared to the highly variable and cold last glacial period (Fig. S6).

**Figure 3:**
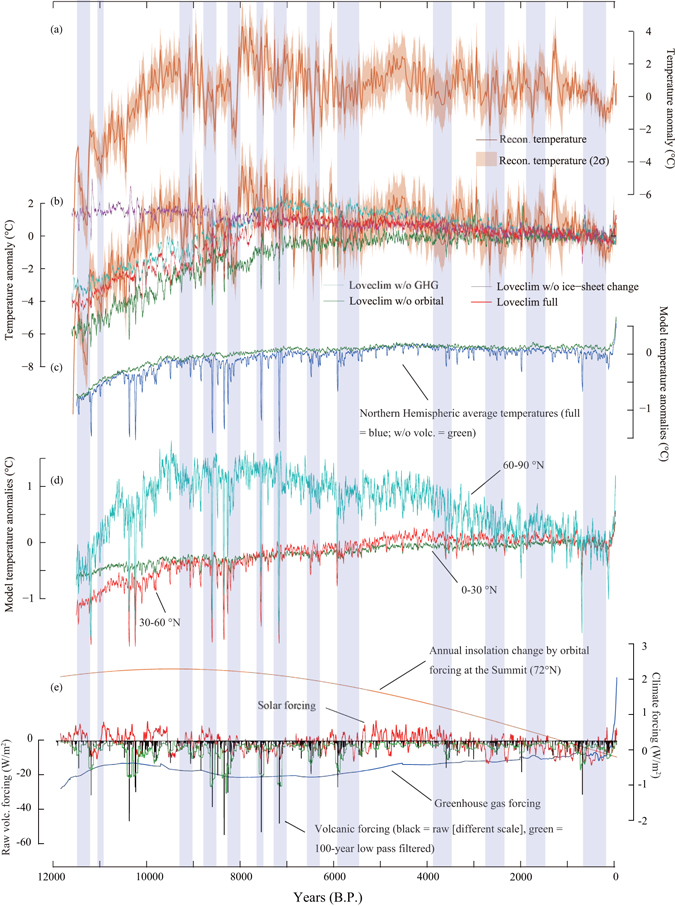
Holocene climate forcing. (**a**) Reconstructed Greenland temperature. (**b**) Reconstructed and modeled Greenland temperatures over the Holocene from various climate model experiments. Experiments without volcanic and solar forcing that exhibit long-term trends similar to that of full forcing are not shown for the sake of simplicity. (**c**) Modeled NH average temperatures with full forcing (blue) and without volcanic forcing (green) both relative to the average of the past 1000 years from the full forcing run. (**d**) Modeled high (60–90°N), middle (30–60°N), and low (0–30°N) latitude average temperatures. (**e**) Annual insolation at 72°N (orange)^63^ and solar activity (red)^31^, GHG (blue; include CO_2_, CH_4_, and N_2_O, Methods) and volcanic forcing as raw (black) and 101-year RMs (green) (Methods). Note that the raw volcanic forcing has a different scale. Values are relative to averages of the past 1,000 years. Model outputs are from individual runs and smoothed by 21-year RMs. Blue shades are the Greenland cold episodes as in Fig. 2.

After an abrupt warming at the end of the Younger Dryas period, Greenland temperature gradually decreased toward the Holocene temperature minimum at 11,271 ± 30 years B.P. (9.2 ± 1.5 °C colder than the recent decades; Preboreal Oscillation), which was followed by another abrupt warming (Figs 2 and 3)^16^. From 11,000 years B.P. to 9,500 years B.P., the temperature kept rising at a rate of 3.7 ± 0.1 °C per 1,000 years (Figs 2 and 3). At 9,200 years B.P., Greenland experienced a rapid cooling (9.2 ka event)^17^. Afterwards, the temperature gradually decreased with occasional large drops (Figs 2 and 3). Then, Greenland experienced the largest hemispheric-wide negative temperature excursion during the Holocene around 8,200 years B.P. (8.2 ka event)^18,19^.

After the 8.2 ka event, Greenland temperature reached the Holocene thermal maximum with the warmest decades occurring during the Holocene (2.9 ± 1.4 °C warmer than the recent decades) at 7960 ± 30 years B.P. Since then, the Summit temperature record exhibits a long-term cooling at a rate of 0.19 ± 0.01 °C per 1000 years towards the present (1.5 ± 0.1 °C cooling in total), agreeing with other estimates from melt layer frequency (1.3 °C) in GISP2 ice core^20^ (Fig. 2b) and from the western Arctic (1.6 ± 0.8 °C; an average estimate of western Arctic cooling)^21^. Greenland experienced a temperature minimum around 5,500 years B.P. Afterwards, the temperature shows a slight increase for 1,500 years (Mid-Holocene Optimum), followed by a cooling into the Little Ice Age with occasional bumps (Fig. 2). From the coldest decades in the Little Ice Age (1740–1780 C.E.) to the recent decades, Greenland Summit temperature increased by 2.9 ± 0.9 °C.

**Figure 4:**
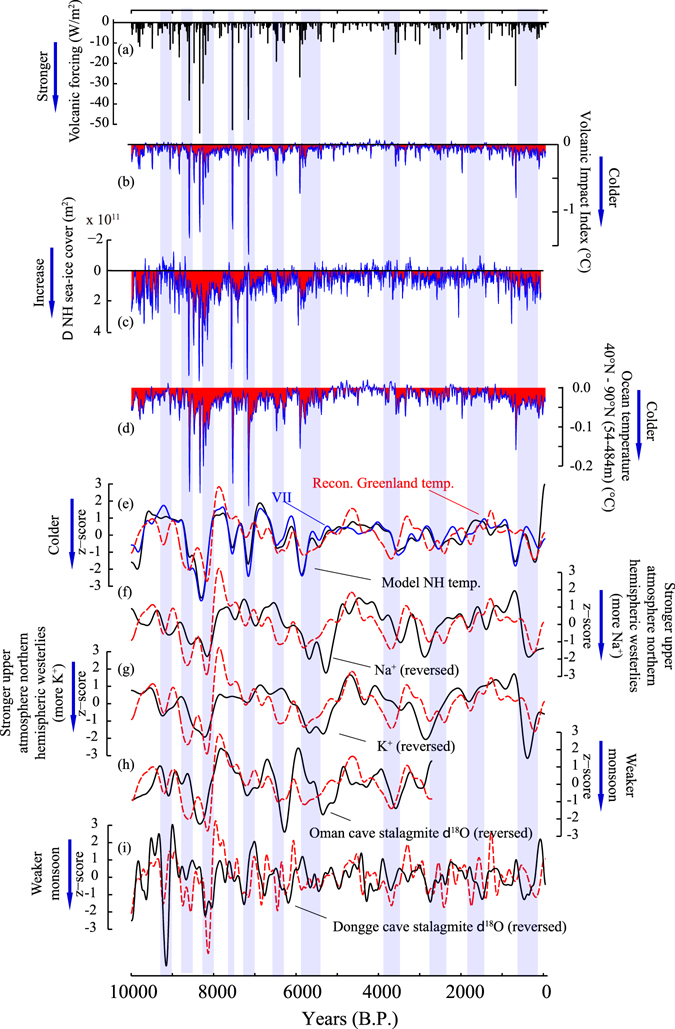
Centennial to millennial-scale volcanic impacts on climate over the past 10,000 years. (**a**) Raw volcanic forcing, (**b**) Volcanic Impact Index (VII), the difference between two curves in Fig. 3c. (**c**) ΔNH sea-ice cover (y-axis is reversed) is calculated as differences between experiments with full forcing and without volcanic forcing (ensemble means) in 21-year RMs, (**d**) as in **(c**) but for ocean temperature (54–484 m) in 40°N–90°N. Red dotted lines in (**e**–**i**) show bandpass-filtered Greenland temperatures, which are compared to (**e**) modeled NH average temperatures (Methods), and VII, (**f**) Na^+^ ion in GISP2 (a proxy for atmospheric circulation)^22^, (**g**) K^+^ ion in GISP2 (proxy for atmospheric circulation)^22^, (**h**) Oman cave stalagmite δ^18^O^1^, (**i**) Dongge cave stalagmite δ^18^O^64,65^. Different bandpass filters were applied for (**e**,**f**,**g**,**h**) and (**i**) with 400–4000 year bands and 200–2000 year bands after linear detrending, respectively (Methods). Red areas in (**b**–**d**) indicate periods under relatively strong volcanic influence. Blue shades are the Greenland cold episodes as in Fig. 2.

The centennial to millennial-scale variability of the reconstructed Greenland temperature is significantly correlated with Na^+^ and K^+^ ion concentrations^22,23^ from the GISP2 ice core, which are proxies for atmospheric circulation, as well as with the Oman and Dongge cave stalagmite δ^18^O records, proxies for monsoon activity, thus indicating that the reconstructed Greenland temperature variations contain hemispheric climate signals^3,23^ (Methods; Fig. 4f–i). This is consistent with the observation that Greenland temperature generally followed proxy-based Northern Hemisphere (NH) average temperature changes over the past 2,000 years^4^.

To investigate possible causes of the temperature variability, we conducted a suite of climate model experiments with different sets of orbital, volcanic, solar, greenhouse gasses (GHG; CO_2_, CH_4_, and N_2_O; Methods) forcings (Fig. 3e) as well as prescribed ice-sheet changes (i.e., ICE6G^24^; ice covered area, topography, and ice-sheet thickness) using the climate model of intermediate complexity, LOVECLIM^25^ (Methods; Fig. 3). Volcanic forcing is reconstructed using the GISP2 sulfate record^22^, which agrees well with multi-core reconstructions^26^ for centennial to millennial-scale variability during the overlapping period of the past 2,500 years (Methods; Fig. S7).

The experiments that include all forcings generally reproduce long-term Greenland temperature variations quite well with a cooling of 0.17 °C/1000 years since 7000 years B.P. (Fig. 3b). Five additional experiments were conducted with sets of forcings that excluded one out of the GHG, orbital, volcanic, solar, and ice-sheet forcings at a time (Fig. 3b–d). An experiment with fixed present-day ice-sheet configuration indicates that the temperature rise from the beginning of the Holocene to 7,000 years B.P. was the result of the retreating Laurentide ice-sheet^27^, while the thinning of ice-sheet (~70 m)^24^ played a minor role on the middle to late Holocene cooling (Fig. 3; Fig. S8).

In these simulations, meltwater inputs from retreating Northern Hemispheric ice-sheets were not taken into account. Particularly, through changes in ocean heat content, the Younger Dryas could have had an impact on temperature trends in the early Holocene (12,000–10,000 yrs B.P.). In addition, through weakening of the Atlantic Meridional Circulation and associated reduction in poleward oceanic heat transport, deglacial meltwater fluxes could have influenced the Northern hemispheric climate until 7,000 years B,P., and particularly during the 8.2 ka B.P. event^28^.

Two numerical experiments exhibit significantly different temperature trends from the middle Holocene to the present (Fig. 3b). From 7,000 years B.P. to the present, the experiment without orbital forcing displays a warming trend, whereas the simulation without GHG forcing overestimates the cooling trend. Therefore, the decreasing temperature trend from the middle to the late Holocene likely results from orbital forcing, but 43% of the orbitally induced cooling was compensated by the increasing GHG forcing (Fig. 3b). The reconstructed Greenland temperature and modeled NH average temperature are significantly correlated (*r* = 0.52, *p* < 0.01) on a centennial to millennial scale (400–4000 year band; Fig. 4e) over the past 10,000 years. The amplitude of temperature variability in simulated NH average temperature is small, but increases from low to high latitudes with amplified noises (Fig. 3d). Over 80% of the simulated hemispheric variability can be explained by volcanic forcing, as the millennial-scale variability (400–4000 year band) of NH temperature and volcanic impact index (VII = “NH average temperatures from full forcing experiments” minus “NH average temperature from no-volcanic forcing experiments” as in Figs 3c and 4b) are highly correlated (*r* = 0.90, *p* < 0.001) (Fig. 4e). Importantly, the VII has a significant correlation with the reconstructed Greenland temperature (*r* = 0.48, *p* < 0.01; Fig. 4e). As volcanic eruptions should not a-priori be related with changes in meltwater forcing, volcanic impacts on the variability of Greenland temperature do not exclude possible meltwater impacts (e.g., the 8.2 ka event^28^).

Recent studies indicate that large volcanic eruptions separated by several decades could induce decadal to centennial cooling in the northern hemisphere (e.g., Little Ice Age) by sea-ice/ocean feedbacks long after volcanic aerosols are removed from the atmosphere^26,29^. In our model experiments, volcanic impacts on climate show clear multi-decadal to millennial variabilities (Fig. 4), although the millennial signals are smaller than those found in the reconstructed temperatures (Fig. 3). The reduced magnitude of the millennial-scale signals in the model could partly be explained by the lack of a cryosphere component and thus potential positive feedbacks between the ice-sheet and ocean-atmosphere system.

In our simulations, a single large volcanic eruption induces rapid atmospheric and ocean surface cooling coupled with sea-ice expansion, which lasts about 16 years. However, slightly lower temperatures persist at high northern latitudes for a century or longer owing to decreased ocean temperatures, which affect ocean surface and sea-ice formation (Fig. 4a–d). Furthermore, a series of large volcanic eruptions (e.g., 9000–8000 years B.P. and the Little Ice Age) produces cold periods of centennial to millennial duration (Fig. 4a–d), which coincide with the reconstructed Greenland temperature decreases, stronger polar atmospheric circulation ([K^+^] and [Na^+^] in GISP2), and weaker Asian monsoon activity (Fig. 4e–i).

The period between 8,600 and 8,000 years B.P. featuring the most intense volcanic activity is also the one corresponding to strong meltwater outbreak from the Laurentide ice sheet^28^ (Fig. 4). The beginning of the cooling started earlier than the rapid drop in δ^18^O_ice_ at 8,200 years B.P. (the 8.2 ka event; Figs 2 and 3). The pre-8.2 ka cooling has been found in many summer temperature proxies^18^, consistent with volcanically induced summer cooling. Therefore, the 8.2 ka cooling event^18^ occurred during a longer-term cold period attributable to volcanic origin, which could have enhanced the sensitivity to meltwater outbreaks from the Laurentide ice sheet. We also note that the mid-Holocene Optimum (5,500–4,000 years B.P.) is characterized by a lack of large volcanic eruptions (Figs 3 and 4). After a period of intensive volcanism and associated cooling (6,000–5,800 years B.P.), a gradual return to a volcanically unperturbed state can be found in modeled 60–90°N average temperatures and in the reconstructed Greenland temperatures. Volcanic activity intensifies again towards the Little Ice Age. Only during the mid-Holocene Optimum were the ocean temperatures in the volcanically unperturbed state (Fig. 4), suggesting that the Holocene climate was mostly under the persistent influence of volcanic activity that is consistent with our earlier studies for the past 4000 years^30^.

Comparisons between the reconstructed Greenland temperatures, reconstructed solar irradiance (TSI)^31^, and model results indicate that solar variability did not play a persistent role in driving millennial-scale variability in Greenland. Nevertheless, solar variability had significant negative imprints on centennial temperature changes especially during the colder periods of the early and late Holocene (stronger solar activities induced colder temperatures in Greenland, or vice versa; Fig. S9) consistent with earlier studies^4,32^. Notably, periods of stronger solar activity in the early Holocene coincide with several rapid cooling periods (e.g., the Preboreal Oscillation and the 8.2 ka event; Fig. S9), indicating that abrupt climate change events during the Holocene may result from changes in several forcings (volcanic, solar, and meltwater inputs), with possible synergetic effects.

We reconstructed the seasonally unbiased and physically constrained Greenland temperature over the Holocene. In contrast to δ^18^O_ice_, the reconstructed Greenland temperature exhibits clear centennial to millennial-scale variability, which is reproduced by climate model simulations including volcanic forcing. Large volcanic eruptions had sustained impacts on climate and people over the past 2,500 years^26^. Clearly, the volcanic impacts were not limited to the recent millennia, but persisted throughout the Holocene with possible important roles on human societal development.

## Methods

### Statistics

To evaluate relationships of two time series, we applied the Pearson correlation coefficient. We consider autocorrelation of the time series to evaluate the significance of correlations by estimating the effective degree of freedom that depends on the effective decorrelation time^33,34^. Correlations or any statistics with >95% confidence levels are considered as significant. Reported uncertainty ranges are 2σ standard deviation unless otherwise stated. Z-score is a measure of a time series having mean zero and standard deviation of one. For filtering of the time series, we used a MATLAB function, “butter”.

### Chronology

We used the Greenland ice core chronology (GICC05), which is based on annual layer counting from multiple cores, synchronized with common volcanic layers^35,36^. Recent studies indicated that GICC05 has slight offsets (up to 70 years^37^ in the early Holocene) from true ages, or more precisely, from dendrochronologically-inferred calendar ages^26,37,38^. Therefore, we correct the offsets using the coefficients from Muscheler *et al*.^37^. The corrected chronology thus has a common time scale with ^14^C-dated paleo records. For gas age calculations, we used a firn-densification and heat diffusion model^39^ for the entire Holocene as an extension of the past 2,100 year calculation from our earlier study^4^. The agreement of timing of climate changes (e.g., the 8.2 ka event and Preboreal Oscillation) between δ^18^O_ice_ and the reconstructed temperature confirms the validity of the estimated gas ages.

### Temperature calculation

Oxygen isotopes of ice (δ^18^O_ice_) have been widely used as a temperature proxy^14,15^, but δ^18^O_ice_ is influenced for example by seasonal changes of accumulation and water vapor source regions^15,40,41^. Borehole temperature reconstructions are a robust method to infer past temperatures, but their resolution decreases rapidly towards earlier ages^42^. Therefore, we have been developing a method to overcome drawbacks of existing temperature proxies using argon and nitrogen isotopes within occluded air in ice cores^4,7–9,43^.

The temperature calculation generally follows earlier studies for the past few millennia^4,7^ with slight modifications for a longer period of the Holocene. We used argon and nitrogen isotope data over the Holocene from the GISP2 ice core^9^. Argon isotopes data requires a minor correction using δAr/N_2_ data to compensate for possible fractionation during bubble close-off between open and closed pores^7,8^. However, δAr/N_2_ data exhibit anomalous enrichments at brittle ice depths of a brittle zone (9,000–6000 years B.P.)^9,43^, which prohibited us from calculating the surface temperature through the Holocene until now. As we gained understanding of the δAr/N_2_ fractionation in the firn layer^43^, it became possible to estimate pre-coring δAr/N_2_ values^43^ at the brittle zone by using accumulation rate^44,45^ and δ^18^O_ice_^46^ data. For the argon isotope correction, we used δAr/N_2_ data^9^ for the past 6,000 years and the estimated δAr/N_2_ for the earlier part of the Holocene. However, we found that the surface temperature reconstruction by correcting argon isotopes by a constant value (i.e., without δ^18^O_ice_ and accumulation rate) is essentially the same as the surface temperature reconstruction with argon isotopes corrected by the estimated δAr/N_2_ using the δ^18^O_ice_ and accumulation rate. For the coefficient of argon isotope correction with δAr/N_2_, we applied the value (0.0073‰/‰) that was used for the temperature reconstruction over the past 4,000 years^7^.

Temperature gradients [Δ*T* (°C) = (δ^15^N-δ^40^Ar/4)/*k*] (Fig. S2) between the top and bottom of the firn layer^6^ can be obtained from nitrogen and the corrected argon isotope data with the coefficient (*k*) obtained from laboratory experiments^47,48^. The Δ*T* data were splined^49^ with a 51-year cutoff period (Fig. S3). Then, the data were again resampled for the sampling ages and used for the following calculations. Surface temperatures were calculated using a firn densification/heat diffusion model^39^ by integrating the Δ*T*s^4,7,8^. For a long integration of Δ*T*, it is important to have precise Δ*T* values as small errors could lead to drifts on the calculated surface temperatures during the integration. We circumvented the drifts by allowing slight constant shifts in Δ*T* by minimizing the difference between the observed and modeled δ^15^N. The Δ*T* integration was performed by dividing the Holocene into nine sections (about 1500-year intervals) including the sections of different measurement periods (e.g., different extraction setups^9^) to minimize the drift. We tested different sections and found little differences in the calculated surface temperatures. Observed and modelled Δ*T*s agree within their uncertainties (Fig. S3).

The calculation started at 50,000 years B.P. to take a memory effect of temperature in the ice-sheet^39^ into account. From 50,000 to 20,000 years B.P., δ^18^O_ice_ was used as temperature after the calibration^45,46^. For the later period to the beginning of the Holocene (20,000 to 11,460 years B.P.), a temperature reconstruction from δ^15^N was used^50^. For both periods, slight constant shifts for prescribed temperatures were allowed to fit with borehole temperatures during the following minimization procedure. Surface temperature calculations are conducted automatically to minimize the differences between the modelled and observed δ^15^N and borehole temperature profiles with parameters (A, B) using a MATLAB function, “fminsearch” (Nelder-Mead simplex method)^51^. In total, 843 Greenland temperature time series were generated from different realizations of argon and nitrogen isotopic values produced within estimated errors^4^. For one realization, about 360 runs from 50,000 years B.P. to present were necessary with different values for each parameter. We further selected 646 realizations that have root mean square deviations of <0.0185‰ for the difference between the modeled and observed δ^15^N and <0.2 °C for the borehole temperature profiles. The 646 realizations were used to calculate mean and standard deviation of the reconstructed Greenland temperature. Comparisons between the calculated surface temperatures and raw Δ*T* variations (calculated from δ^40^Ar not corrected for argon loss) indicate that most of the variability in the reconstructed temperature originates from the variability in raw Δ*T* (Fig. S2). Argon corrections or surface temperature calculations only slightly influence the variability in the calculated surface temperature over the past 10,000 years (Fig. S2)^4^.

The temperature reconstruction from 1941 to 1993 C.E. was done using a borehole temperature inversion technique^4^, and a slight temperature shift at 1941 in Fig. 1 is due to the change in method. We also note that temperature calculations using only δ^15^N^40,41^ produce somewhat different surface temperature histories. To capture small temperature changes such as those in the Holocene, high-precision nitrogen and argon data^9^ and calculated temperature signals Δ*T* are critical for the surface temperature calculation.

In Fig. 2, the reconstructed Greenland temperatures are compared with average δ^18^O_ice_ (GRIP, NGRIP, and GISP2). The average δ^18^O_ice_ has a temperature sensitivity of 0.36 permil/°C (geometric regression), and the correlation coefficient between the average δ^18^O_ice_ and temperature is *r* = 0.80 (*p* = 0.01). The reconstructed Greenland temperature vs δ^18^O_ice_ GRIP, NGRIP, and GISP2 has correlations of *r* = 0.76 (*p* = 0.01), 0.76 (*p* = 0.02), and 0.71 (*p* = 0.01), respectively. In Fig. 4, bandpass-filtered (400–4000 year bands) Greenland temperatures are compared with modeled NH average temperatures (*r* = 0.52, *p* < 0.01), VII (*r* = 0.49, *p* < 0.01), Na^+^ ion in GISP2 (*r* = −0.49, *p* = 0.01), K^+^ ion in GISP2 (*r* = −0.51, *p* < 0.01), Oman cave stalagmite δ^18^O (*r* = −0.44, *p* = 0.02), Dongge cave stalagmite δ^18^O (*r* = −0.28, *p* < 0.01) that exhibits a significant correlation with the Greenland temperature in shorter periods (200–2000 year bands). The Dongge cave record has a less significant correlation (*r* = −0.23, *p* = 0.12) on 400–4000 year bands presumably because it is less influenced by North Atlantic climate. The bands were chosen to remove long term trends (e.g., orbital scale variation) and reduce noise.

### Climate model experiments

We employed a three-dimensional Earth system model of intermediate complexity, LOVECLIM version 1.3^25^. The atmospheric component is ECBilt2, a T21, 3-level quasi-geostrophic model, fully coupled to the ocean general circulation model, CLIO3 (3° by 3° horizontal resolution, 20 vertical levels) and to the vegetation model, VECODE. To conduct transient experiments of the Holocene period, volcanic, solar, greenhouse gas and orbital^52^ forcings were applied with variable ice-sheet configurations (ICE6G)^24^. For the solar forcing, we used a reconstruction of total solar irradiance (TSI) for the Holocene from tree-ring ^14^C data^31^. The greenhouse gas forcing includes CO_2_^53–55^, CH_4_^56^, and N_2_O^57^, and concentration data are used as inputs in the model. The ice-sheet changes include extents, thickness (e.g., Greenland Ice-sheet), and ice covered area from the ICE 6 G database^24^. To precondition the ocean before the Holocene integration, an equilibrium simulation was conducted for 2,700 years with parameters for 20,000 years B.P., after which the model was transiently integrated towards the beginning of the Holocene with variable ice-sheets, greenhouse gas and orbital forcings (pre-Holocene run). A suite of Holocene experiments, initiated from 12,000 years B.P., were then performed with different sets of forcings. The initial conditions (a, b, c, and d) were derived from the end of the pre-Holocene run (12,000 years B.P.), and dates going back approximately 100 years apart from the end of the pre-Holocene run. We conducted four experiments with full forcings (initial conditions = a, b, c, and d), four experiments with full but without volcanic forcing (initial conditions = a, b, c, and d), and four experiments with full but without greenhouse gas, solar, orbital, or ice-sheet forcing.

### Volcanic forcing

Volcanic forcing is generated from a sulfate concentration record of GISP2^22^ and is an extension of the volcanic forcing generated for the study over the past 4,000 years^30^. The method^30^ generally follows a study by Gao *et al*.^58^. The selected volcanic peaks are set to decay with an e-folding time of 1 year, approximating sulfate aerosol removal processes in the atmosphere^30,59^. The GISP2 sulfate record has been used as a proxy for volcanic eruptions in numerous studies^3,60,61^. However, to our knowledge, this is the first study to use the record to produce volcanic forcing and run a climate model through the Holocene. The sulfate signals are linearly related to volcanic forcing. It has been indicated that sulfate loading in the atmosphere may become increasingly less effective to radiative forcing^62^. Therefore, linear relation may overestimate the size of the large eruptions.

We compared the volcanic forcing with more complete volcanic forcings from several studies using multi-ice cores^26,58^ (Fig. S7). For the past 1,500 years, the volcanic forcings from this study and Sigl *et al*.^26^ (Fig. S7b,c) have a correlation of *r* = 0.78, *p* = 0.0018, while this study vs. Gao *et al*.^58^ has a correlation coefficient *r* = 0.86, *p* = 0.0017, and Gao *et al*.^58^ vs. Sigl *et al*.^26^ have a correlation of *r* = 0.86, *p* < 0.001. Therefore, over 70% of the total variance of volcanic forcing from multiple cores (Gao *et al*.) can be obtained by the single core (GISP2). For the past 2,500 years, this study and Sigl *et al*.^26^ display a slightly lower correlation of *r* = 0.69, *p* < 0.001, indicating that the reconstructed volcanic forcing obtained in this study well captures centennial to millennial trends of more complete volcanic forcings (Sigl *et al*.^26^ and Gao *et al*.^58^). In addition, we note that during the periods of frequent large eruptions such as in the past 1,000 years, volcanic forcing reconstruction from a single core performs better than the periods of less frequent eruptions. The reconstructed volcanic forcing sometimes misses eruptions when compared with more complete reconstructions in raw data^26^ (Fig. S7). Therefore our reconstruction is likely to be a lower-band estimate of true volcanic forcing. Also, we note that the strength of our volcanic forcing is located in the middle between Sigl *et al*.^26^ and Gao *et al*.^58^ reconstructions (Fig. S7), although the strength of true volcanic forcing is still under debate. In LOVECLIM, the volcanic forcing is implemented through anomalies in solar irradiance at the top of the atmosphere.

## Electronic supplementary material

Extended Data Figure 1 to 9

Supplemental information: Data
