# Author Correction: Volcanic influence on centennial to millennial Holocene Greenland temperature change

**DOI:** 10.1038/s41598-018-21307-y

**Published:** 2018-03-06

**Authors:** Takuro Kobashi, Laurie Menviel, Aurich Jeltsch-Thömmes, Bo M. Vinther, Jason E. Box, Raimund Muscheler, Toshiyuki Nakaegawa, Patrik L. Pfister, Michael Döring, Markus Leuenberger, Heinz Wanner, Atsumu Ohmura

**Affiliations:** 1 Climate and Environmental Physics, University of Bern, 3012 Bern, Switzerland; 2 Oeschger Centre for Climate Change Research, University of Bern, 3012 Bern, Switzerland; 3 Climate Change Research Centre and PANGEA Research Centre, University of New South Wales, New South Wales, 2052 Australia; 4 ARC Centre of Excellence for Climate System Science, New South Wales, Sydney Australia; 5 Centre for Ice and Climate, Niels Bohr Institute, University of Copenhagen, 2100 Copenhagen, Denmark; 6 Geological Survey of Greenland and Denmark, 1350 Copenhagen, Denmark; 7 Department of Geology, Quaternary Sciences, Lund University, 22362 Lund, Sweden; 8 Meteorological Research Institute, Tsukuba, 305-0052 Ibaraki Japan; 9 Institute for Atmospheric and Climate Science, Swiss Federal Institute of Technology ETH Zurich, 8092 Zurich, Switzerland; 10 Present Address: Renewable Energy Institute, Minato-ku, 105-0003 Tokyo Japan

Correction to: *Scientific Reports* 10.1038/s41598-017-01451-7, published online 03 May 2017

The original Supplementary Information file published with this Article was incorrect. This version included errors in the Greenland temperatures, and the ice core depth information was omitted. The correct Supplementary Information file now accompanies the Article.

