# Supplementary material for: Volcanic influence on centennial to millennial Holocene Greenland temperature change
Source: Sci Rep. 2017 May 3;7:1441. doi: 10.1038/s41598-017-01451-7 (PMC5431187; doi:10.1038/s41598-017-01451-7)
Supplement: Supplementary file 1 — Extended Data Figure 1 to 9 [file 41598_2017_1451_MOESM1_ESM.pdf]

Supplementary Information for

Volcanic influence on centennial to millennial Holocene Greenland temperature change

Takuro Kobashi*, Laurie Menviel, Aurich Jeltsch-Thömmes, Bo M. Vinther, Jason E. Box, Raimund Muscheler, Toshiyuki Nakaegawa, Patrik L. Pfister, Michael Döring, Markus Leuenberger, Heinz Wanner, Atsumu Ohmura

Correspondence to: kobashi@climate.unibe.ch

This file includes:

References

Figs. S1-9

Supplementary Dataset (1 excel file)

References

1 Kobashi, T., Severinghaus, J. P. & Kawamura, K. Argon and nitrogen isotopes of trapped air in the GISP2 ice core during the Holocene epoch (0-11,600 B.P.): Methodology and implications for gas loss processes. *Geochim. Cosmochim. Ac.* **72**, 4675-4686 (2008).

2 Enting, I. G. On the Use of Smoothing Splines to Filter Co2 Data. *J. Geophys. Res.-Atomos.* **92**, 10977-10984 (1987).

3 Severinghaus, J. P., Sowers, T., Brook, E. J., Alley, R. B. & Bender, M. L. Timing of abrupt climate change at the end of the Younger Dryas interval from thermally fractionated gases in polar ice. *Nature* **391**, 141-146 (1998).

4 Alley, R. B. & Koci, B. R. Recent warming in central Greenland? *Ann. Glaciol.* **14**, 6-8 (1990).

5 Clow, G. D., Saltus, R. W. & Waddington, E. D. A new high-precision borehole-temperature logging system used at GISP2, Greenland, and Taylor Dome, Antarctica. *J. Glaciol.* **42**, 576-584 (1996).

6 Kobashi, T. *et al.* Persistent multi-decadal Greenland temperature fluctuation through the last millennium. *Climatic Change* **100**, 733-756 (2010).

7 Sigl, M. *et al.* Timing and climate forcing of volcanic eruptions for the past 2,500 years.

Nature **523**, 543–549 (2015).

8 Gao, C., Robock, A. & Ammann, C. Volcanic forcing of climate over the past 1500 years: An improved ice core-based index for climate models. *J. Geophys. Res.-Atomos.* **113**, D23111, doi:10.1029/2008JD010239 (2008).

9 Roth, R. & Joos, F. A reconstruction of radiocarbon production and total solar irradiance from the Holocene 14C and CO2 records: implications of data and model uncertainties. *Clim. Past* **9**, 1879-1909, doi:10.5194/cp-9-1879-2013 (2013).

10 Reimer, P. J. *et al.* IntCal13 and Marine13 radiocarbon age calibration curves 0-50,000 years cal BP. *Radiocarbon* **55**, 1869–1887 (2013).

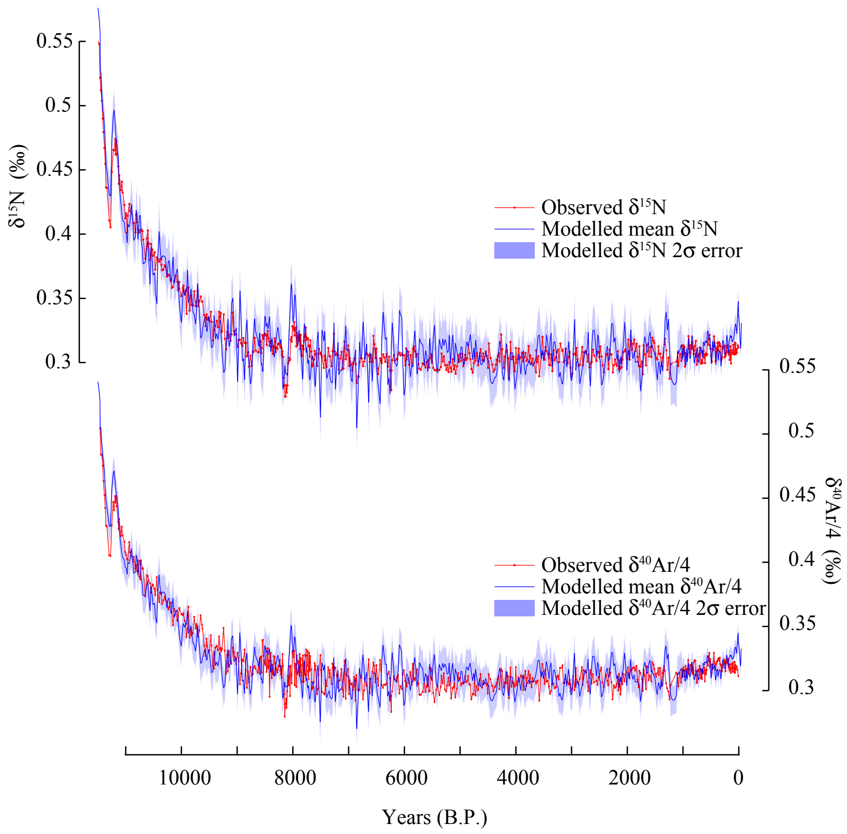

Figure S1. Observed and modeled $\delta^{15}N$ and $\delta^{40}Ar/4$ over the Holocene. Observed $\delta^{15}N$ and $\delta^{40}Ar/4$ are raw data[1].

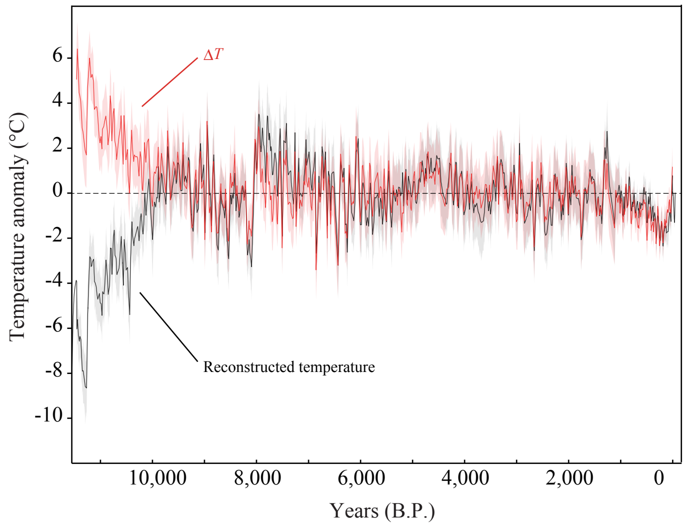

Figure S2. Reconstructed Greenland temperatures and Δ*T* over the Holocene relative to averages over the past 10,000 years. Δ*T*s were calculated from raw nitrogen and argon isotope data (Fig. S1), and splined[2] with a 51-year cutoff period. Error bands are 2σ. A gradual decrease in Δ*T* from the beginning of the Holocene is due to an abrupt warming by ~10 °C at the end of Younger Dryas[3], which created large temperature gradients in the firn. The Δ*T* gradually decreased as surface heat diffused into the ice-sheet. Thereafter, calculated surface temperatures follow Δ*T*, slightly modified by heat diffusion in the firn.

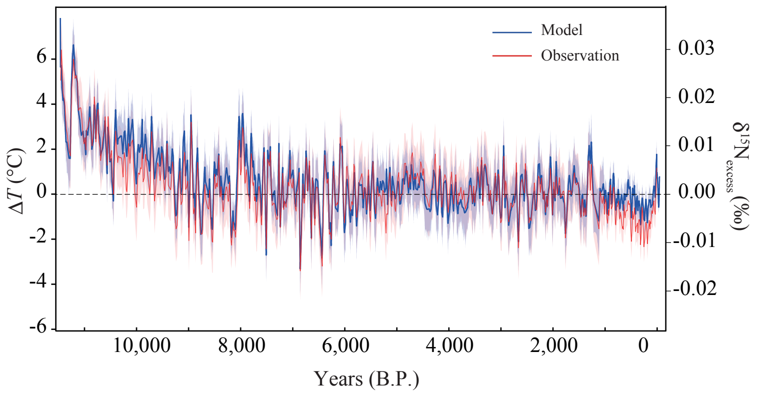

Figure S3. Modeled and observed ΔT and $\delta^{15}N_{excess}$ over the Holocene. Observed data are the same as Fig. S2. Model data is the temperature gradients between the top and bottom of the firn layer during the calculation of the surface temperature. ΔT and $\delta^{15}N_{excess}$ have a relationship of ΔT (˚C) = $\delta^{15}N_{excess}$ (‰) / 0.0047 (‰/˚C).

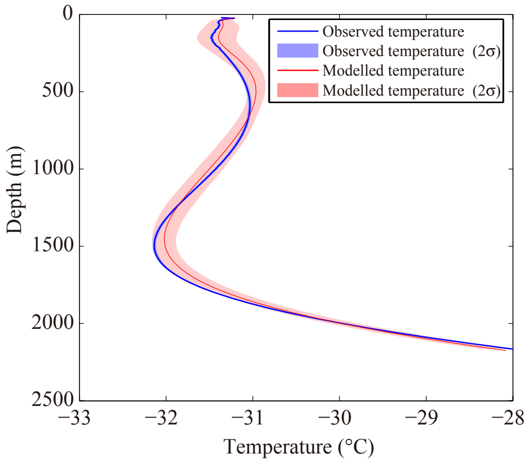

Figure S4. Observed and modeled borehole temperatures for GISP2. Observed borehole temperatures are from Alley and Koci[4] and Clow et al.[5] as used in Kobashi et al.[6].

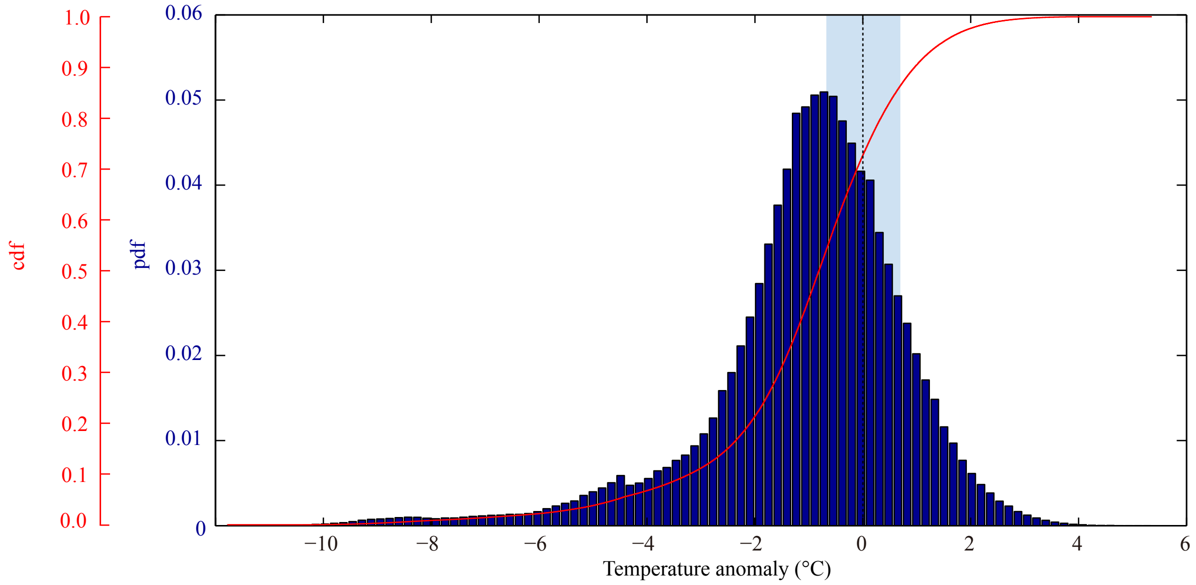

Figure S5. Probability density and cumulative distribution functions (pdf and cdf) of the Holocene Greenland temperatures from 11,550 to -43 years B.P. Temperature anomalies are relative to an average of 1988-2015. A light blue band indicates a range of temperature experienced over the past 28 years in a decadal time scale (11-year RMs), and a dotted line represents 0 °C as an anomaly (i.e., from the average of 1988-2015).

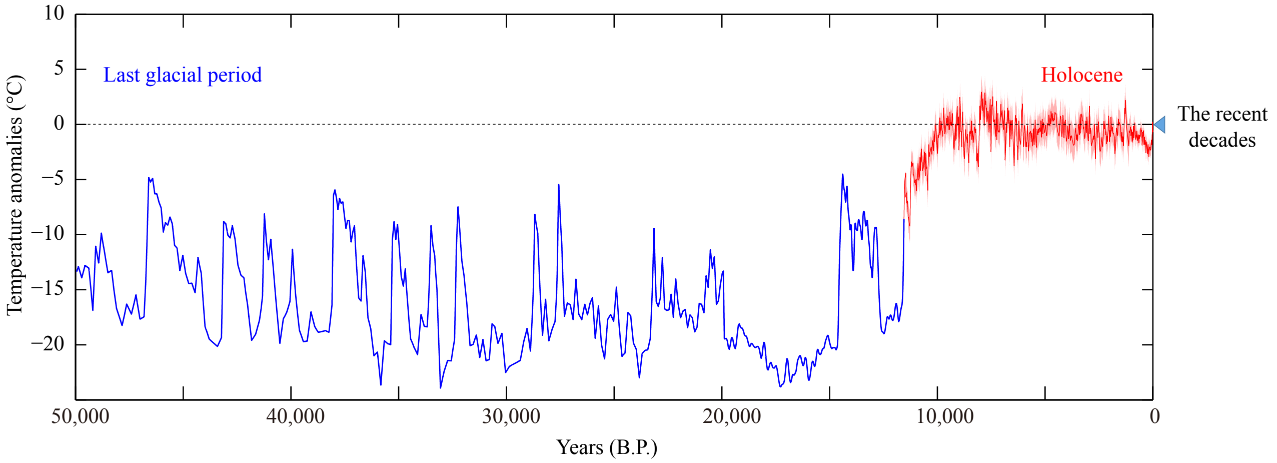

Figure S6. Greenland temperature anomalies over the past 50,000 years relative to an average of 1988-2015. The red line is the reconstructed Holocene Greenland temperature with 2σ error bands. The temperature record during the last glacial period was used for the temperature calculation for the Holocene (see Methods).

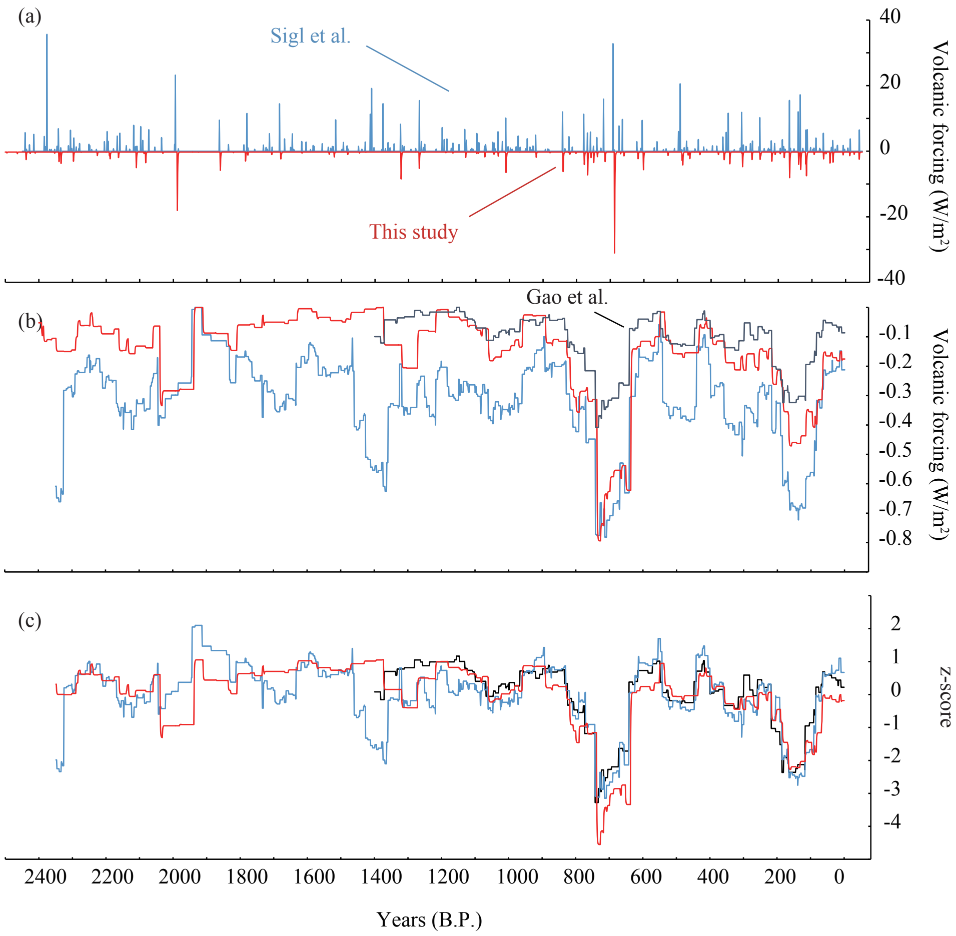

Figure S7. Comparisons of volcanic forcing reconstructions. (a) The red line is volcanic forcing from this study (GISP2), and the blue line is volcanic forcing from NH and tropical eruptions in Sigl et al.[7] using NEEM and NGRIP ice cores. Importantly, the two reconstructions are completely independent. Note that the Sigl data is reversed in its sign for an easier comparison. (b) as of (a) but 101-year RMs are applied. The black line is volcanic forcing for NH from Gao et al.[8] using multiple-ice cores. Their volcanic sulfate data were converted to volcanic forcing following Gao et al.[8]. (c) The volcanic forcings as in (b) are shown in z-score for easier comparisons of their variability.

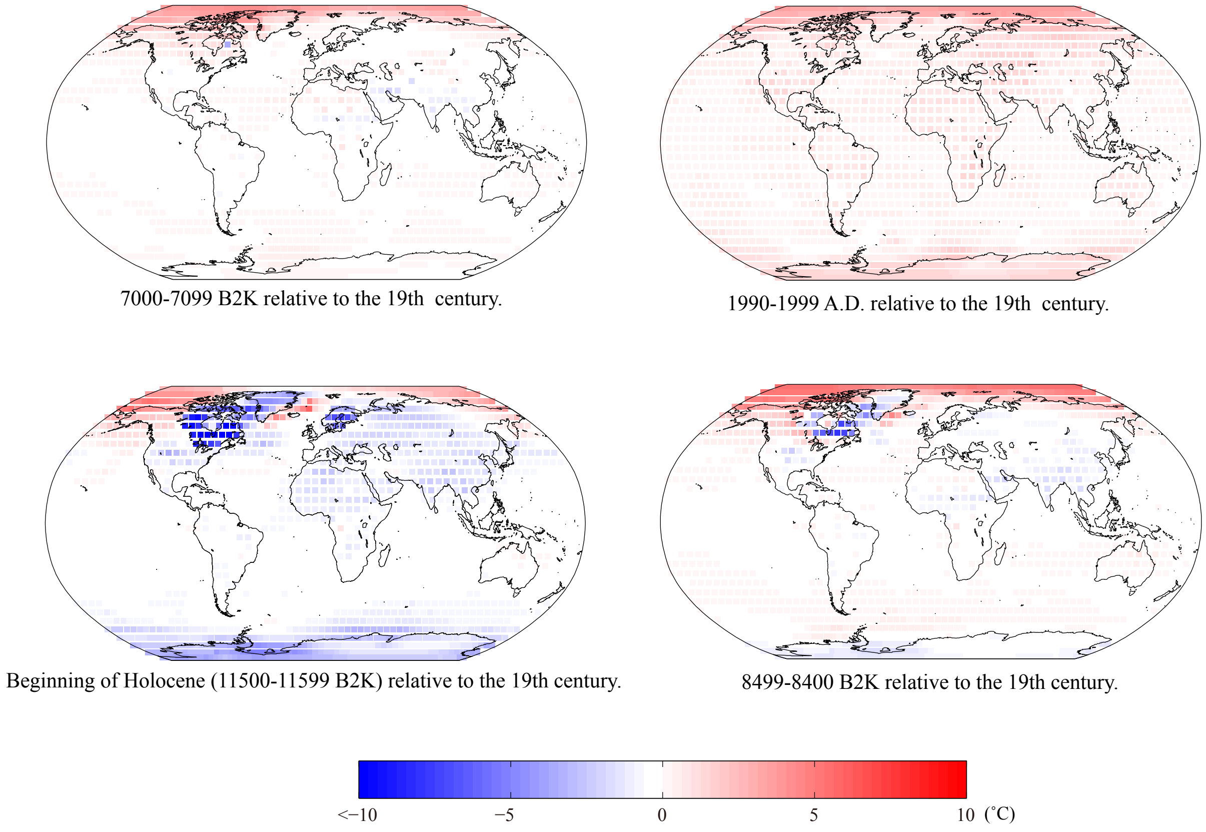

Figure S8. Global temperature anomaly maps in different time intervals during the Holocene relative to averages of the 19th century. B2K represents years before 2000 C.E. To create the map, we used MATLAB R2013a.

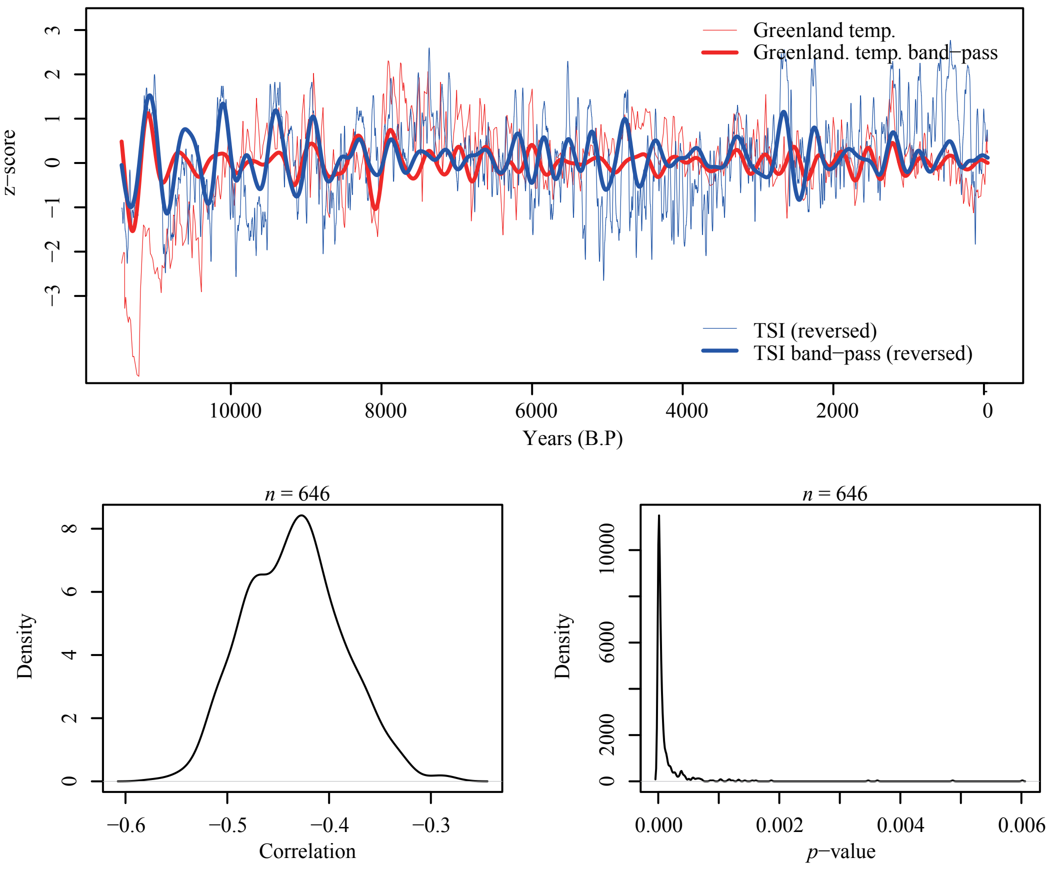

Figure S9. Greenland temperatures and solar activity. Top: Greenland temperature and reconstructed total solar irradiance (TSI)[9] in z-score with a band-pass filter (300-750 years). Bottom left: probability density function of the correlations between the TSI[9] and 646 realizations of Greenland temperatures in filtered data. Bottom right: those of p-values. We also confirmed the similar result using ^{14}C residual data[10].
